# Case Report: Interventional therapy for portal venous stenosis caused by systemic vasculitis

**DOI:** 10.3389/fimmu.2022.1005300

**Published:** 2022-10-12

**Authors:** Qiuyu Cai, Bo Wei, Yang Tai, Hao Wu

**Affiliations:** ^1^Department of Gastroenterology, West China Hospital, Sichuan University, Chengdu, China; ^2^Laboratory of Gastroenterology and Hepatology, West China Hospital, Sichuan University, Chengdu, China

**Keywords:** systemic vasculitis, portal venous stenosis, portal hypertension, angioplasty, stent implantation

## Abstract

Systemic vasculitis are multisystem blood vessel disorders. However, Portal venous involvement is extremely rare, which represents a diagnostic and therapeutic challenge due to the heterogeneous nature, a lack of diagnostic criteria and limited effective therapy of vasculitis. We have reported a 48-year-old woman who was previously diagnosed with systemic vasculitis and was treated with prednisone, presented with gastrointestinal (GI) bleeding on admission. Further abdominal contrast-enhanced computed tomography (CT) with three-dimensional reconstruction suggested atrophic left hepatic lobe, enlarged spleen, and severe stenosis of main portal vein. Liver biopsy showed no evidence of fibrosis/cirrhosis. To prevent rebleeding, portal venous angioplasty by balloon dilation with collateral varices embolization was performed, and the GI hemorrhage was resolved completely. However, refractory ascites presented 8 months postoperatively. Abdominal CT angiography confirmed the recurrence of portal venous stenosis. Portal venous angioplasty by stent implantation was then performed to treat the portal hypertension (PHT)-related complications. After the intervention, the patient received anticoagulation therapy and continued immunosuppressive therapy. During the 5-year follow-up, the patient did not experience any onset of GI bleeding or ascites. Therefore, portal venous angioplasty with stent placement could be an effective treatment to prevent PHT-related complications when immunosuppression therapy failed.

## Introduction

Systemic vasculitis encompasses a heterogeneous group of autoimmune diseases, with inflammation and necrosis of vascular walls as the main pathological changes (1). The disease occurs in variable ways and usually affects multiple organ systems. Many patients present with non-specific systemic symptoms such as recurrent fever, malaise, weight loss, and night sweats (2). More specific symptoms vary depending on the type, size, and severity of vascular involvement (2). Although portal vein is not one of the organs most commonly affected in systemic vasculitis (3), suspicion should always be kept in mind, especially when specific clinical symptoms appear (4). At present, treatment options of vasculitis include use of glucocorticoids, disease modifying anti-rheumatic drugs (DMARDs), and biologics (5). However, the current therapeutic regiments show limited effect in some cases, and the established vascular lesions are sometimes irreversible (4). Herein, we reported a case of severe irreversible portal venous stenosis due to systemic vasculitis that required interventional therapy for palliation.

## Case presentation

A 48-year-old female was admitted to our emergency department for several episodes of massive hematemesis with melena. The patient suffered from intermittent low fever, myalgia and fatigue in recent one year, which had been diagnosed with systemic vasculitis 4 months ago, and oral prednisone was administrated afterwards until this gastrointestinal (GI) bleeding. There was no symptom of abdominal pain, nausea, vomiting or jaundice before the onset of bleeding. She also denied any chronic liver diseases or receiving any other hepatopancreatobiliary surgeries except laparoscopic cholecystectomy for cholecystolithiasis decades ago.

**Figure 1:**
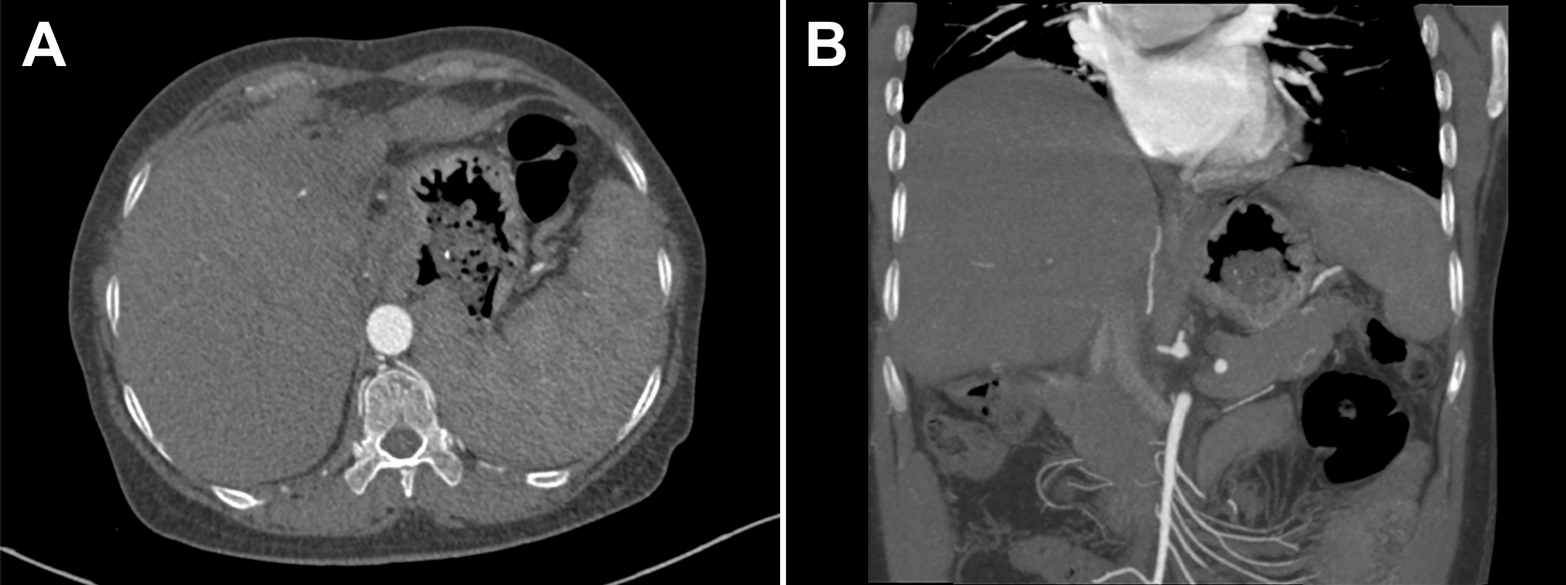
**(A, B)** Abdominal contrast-enhanced computed tomography (CT) showed the atrophic left hepatic lobe and enlarged spleen.

**Figure 2:**
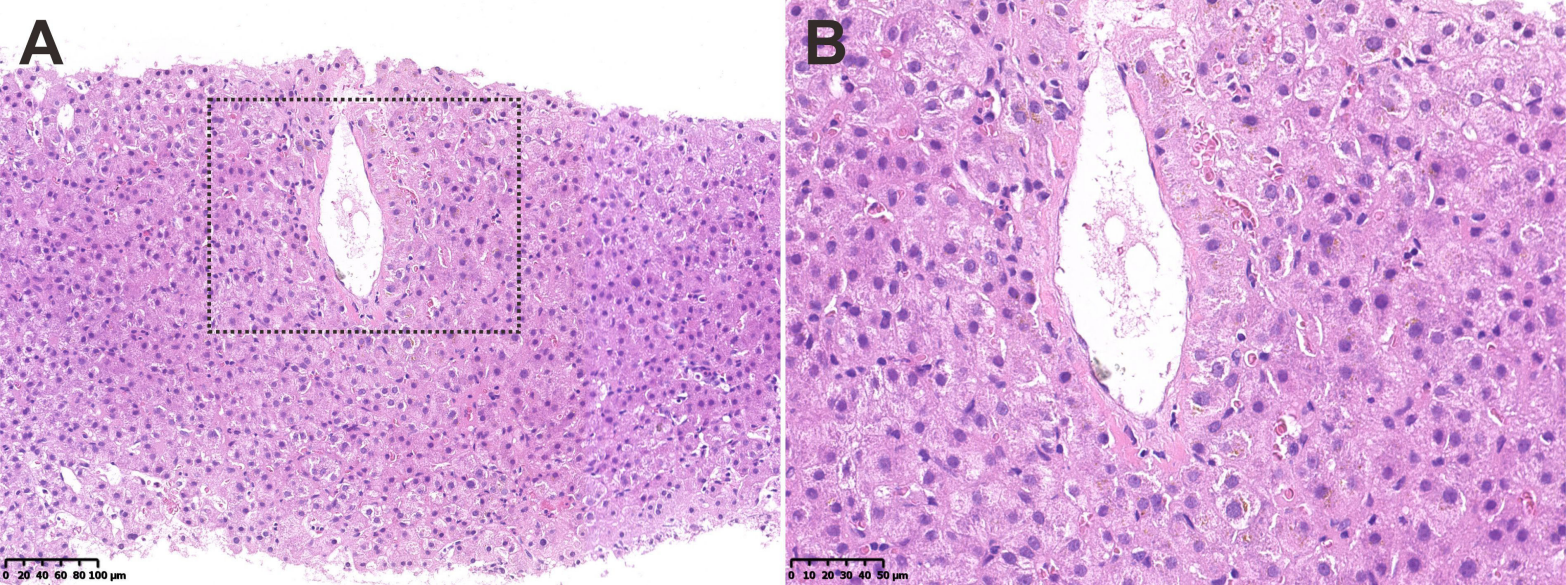
**(A, B)** Liver biopsy showed slight inflammation and cholestasis without fibrosis/cirrhosis.

At the admission, physical examination was unremarkable and laboratory tests showed the decreased hemoglobin (73 g/L, normal, 130-175 g/L), normal leukocyte and platelet counts, normal liver and renal function, increased prothrombin time (16.3 s, normal, 9.6-12.8 s), normal international normalized ratio (INR), and increased D-dimer value (2.78 mg/L FEU, normal < 0.55 mg/L FEU). Upper gastrointestinal endoscopy revealed moderate esophageal varices without active bleeding. Abdominal contrast-enhanced computed tomography (CT) suggested the atrophic left hepatic lobe and enlarged spleen (**Figures 1A, B**). Additionally, liver biopsy showed slight inflammation and cholestasis, but no evidence of fibrosis/cirrhosis was observed (**Figures 2A, B**).

**Figure 3:**
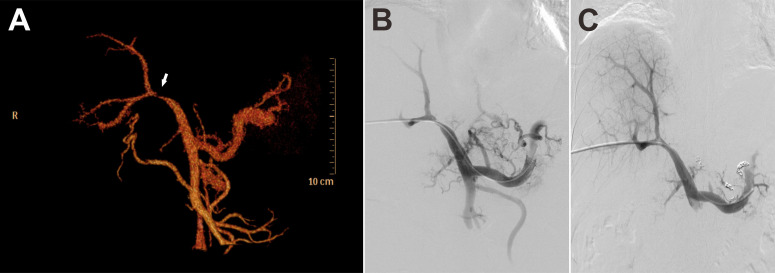
CT angiography (CTA) with three-dimensional reconstruction of portal system demonstrated severe stenosis of main portal vein (**A**, arrow). Portal vein stenosis **(B)** was relieved with portal venous angioplasty by balloon dilation **(C)**.

**Figure 4:**
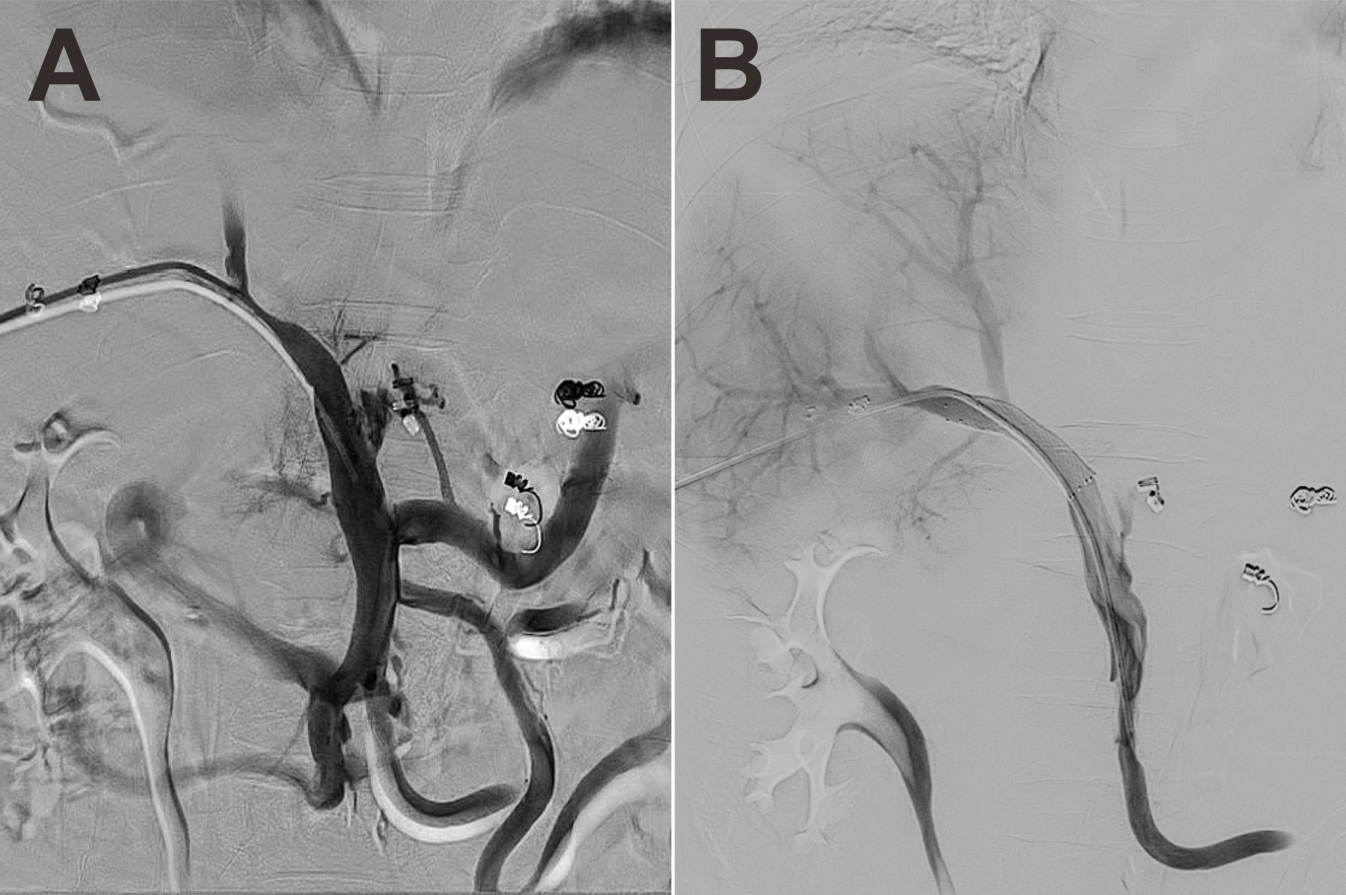
Portal venography showed severe stenosis of the portal vein at the hepatic hilum **(A)**. One 8-mm × 40-mm bare metal stent was placed across the stenosis, allowing rapid portal inflow into the liver and decompression of the large collateral varices **(B)**.

Based on the coexistence of hepatic lobar atrophy and portal hypertension (PHT), vascular disorders of the liver were taken into consideration. Later, CT angiography (CTA) with three-dimensional reconstruction of portal system demonstrated severe stenosis of main portal vein (**Figure 3A**, arrow), which failed to extend branches supplying the left hepatic lobe. Portal venous angioplasty by balloon dilation (8-mm, 10-mm, sequentially) was performed thereafter, with collateral varices embolized by spring coils to prevent rebleeding (**Figures 3B, C**). The pressure gradient across the stenosis decreased from 8 mmHg to 4 mmHg after procedure. Although GI hemorrhage resolved completely and hemoglobin gradually recovered to normal levels, refractory ascites unexpectedly presented 8 months postoperatively. Abdominal CTA confirmed the recurrence of portal venous stenosis. Then portal venography *via* percutaneous transhepatic approach was performed, showing that the portal vein had undergone severe stenosis that nearly occluded it at the hepatic hilum (**Figure 4A**). To relieve the portal venous stenosis, an 8-mm × 40-mm bare metal stent was placed across the stenosis, resulting in rapid portal inflow into the liver and decompression of the large collateral varices (**Figure 4B**). The portal pressure gradient decreased from 18 mmHg to 12 mmHg with stent implantation. The patient received dabigatran anticoagulation therapy and continued immunosuppressive therapy (steroid and cyclophosphamide) after the intervention. During the 5-year follow-up, there was no recurrence of fever, myalgia or fatigue, and the patient did not experience any onset of GI bleeding or ascites.

## Discussion

Systemic vasculitis characterized by autoallergic inflammation of involved vessels, is a spectrum of diseases with diverse clinical presentations, which remains a public health problem worldwide (6). It can cause stenosis/occlusion of any blood vessels, leading to organ ischemia, aneurysm formation and hemorrhage (7). The pathophysiology of systemic vasculitis remains incompletely understood and varies between different forms of vasculitis (5). Immune cells (lymphocytes, macrophages, neutrophils, et al.) and proinflammatory cytokines (IL-1, IL-6, TNF-α, et al.) are assumed to be involved in the inflammatory infiltrates and loss of structural integrity of the vessels (5). Moreover, in some cases, endothelial dysfunction resulted from chronic inflammation promotes thrombotic events, which may interact with immune system cells and amplify the inflammatory cascade (8). However, antigens triggering those process and mechanisms underlying vascular remodeling remain unknown.

By invading the portal system, vasculitis can cause disorders of the portal veins (9), resulting in non-cirrhotic portal hypertension (NCPH). Obliteration of veins can start with endothelial injury caused by inflammatory conditions. As occlusion progresses, the flow of blood was impeded to the liver, and local stasis and low-grade portal hypertension develop. In many cases, portal thrombosis occurs before PHT becomes clinically evident (10). Nevertheless, portal vein is rarely involved at initial presentation of systemic vasculitis. By reviewing the literature, only six cases of NCPH or portal obstruction/stenosis caused by systemic vasculitis have been reported over the past 30 years (**Table 1**). All these cases initially presented with non-specific symptoms such as fever and rash (11–16). The subsequent abdominal pain or elevated transaminase values led to the discovery of portal vein thrombosis (11, 14). Only two patients had significant symptoms of PHT, including hepatosplenomegaly, ascites, and abdominal wall varices (12, 13). One patient experienced atrophy of the left hepatic lobe, and stenosis of portal vein branches was found at autopsy (16).

**Table 1:** The clinical characteristics of patients with NCPH or portal obstruction/stenosis caused by systemic vasculitis.

References	Gender/age	Etiology	Manifestation	PVT	NCPH	Treatment	improvement after Treatment
Wolf *et al.* (11)	F/36	EGPA	Necrotic skin lesions, pulmonary infiltrates, right upper quadrant tenderness	Yes	No	Prednisolone 60 mg qd., low molecular weight heparin, methotrexate	Yes
Natarajan *et al.* (12)	M/48	Churg-Strauss disease	Abdominal distension, fever, ascites, abdominal wall varices	Yes	Yes	Dexamethasone 6 mg tid., tapering dose of prednisolone 1 mg/kg/day, warfarin 3 mg qd.	Yes
Herrera *et al.* (13)	M/9	TA	Recurrent fever, hepatosplenomegaly	No	Yes	Methylprednisolone followed by prednisone, cyclophosphamide, azathioprine, infliximab	No
Abebe *et al.* (14)	M/58	HSP	Rash, nausea, vomiting, abdominal pain, dark stool, hematochezia	Yes	NA	Methylprednisolone 1 g qd. followed by 30 mg q12h.	No
Gelber *et al.* (15)	M/22	Behçet’s Disease	Fever, abdominal pain, weight loss, diarrhea	Yes	NA	NA	NA
Nakazawa *et al.* (16)	M/73	PN	Intermittent fever, abdominal pain, erythema, and myalgia	No, just narrowing of the portal vein	NA	Prednisolone 30 mg/day	No

PVT, portal venous thrombosis; NCPH, non-cirrhotic portal hypertension; EGPA, eosinophilic granulomatosis with polyangiitis; TA, Takayasu arteritis; HSP, Henoch Schonlein purpura; PN, Polyarteritis nodosa; NA, not available.

In this case, the patient had typical clinical manifestations of PHT, including esophageal varices, splenomegaly, and ascites. In addition, fibrosis/cirrhosis and idiopathic portal hypertension were ruled out by a normal liver biopsy. Based on the clinical presentation, a diagnosis of hepatic vascular disorders was suspected. Also, atrophy of the left hepatic lobe was found during abdominal CT scanning. It is well known that hepatic lobar atrophy frequently occurs due to the decrease or remodeling of portal blood supply. The deprivation of portal vein circulation is considered to induce ischemia and infarction of the liver and inhibit the function of hepatocytes in the involved area, leading to the hepatic atrophy (17). Imaging studies found nearly 90% atrophic lobes had ipsilateral portal obstruction (17), which led us to the consideration of portal venous involvement by systemic vasculitis. Severe stenosis of main portal vein from CTA with three-dimensional reconstruction confirmed our speculation. Further venography provides valuable evidence and information for the subsequent interventional therapy option. Thus, once post-NCPH symptoms occurs in systemic vasculitis patients, occlusion/stenosis of portal veins should not be ignored.

Diagnosis of systemic vasculitis remains a challenge due to the heterogeneous nature of vasculitis and a lack of diagnostic criteria, which may hinder early diagnosis (18). Suspicion of vasculitis is an important diagnostic step. Besides, there are developed classification criteria which permit defining subgroup. Anti-neutrophilic cytoplasmic antibody (ANCA) testing, vascular imaging, and biopsy benefit in defining a subgroup of systemic vasculitis and facilitating diagnosis (5). Moreover, the exclusion of secondary causes of vasculitis are needed for diagnosis. Observation over time and therapeutic trials might rule out or improve the probability of the diagnosis when the diagnosis remains uncertain (19). For portal venous involvement alone, radiologic imaging findings may provide the only evidence for the initial diagnosis. CTA with three-dimensional reconstruction is a helpful radiological confirmation. The portal venous phase of angiography plays a crucial role in visualization of the entire portal venous system, revealing the extent of stenosis and existence of other vascular complications, including PVT, CTPV and collateral veins (20).

Glucocorticoids and DMARDs such as cyclophosphamide, methotrexate, mycophenolate mofetil and azathioprine, which have broad impact on both innate and adaptive immunity, have been the core of systemic vasculitis management (21). In addition, biologic therapy including highly cell and cytokine targeted drugs has been applied in the vasculitis arena (1). Although immunosuppressive therapy is the preferred treatment, it occasionally presented limited effect in inhibiting disease progression or alleviating symptoms. Of the six cases we summarized (**Table 1**), only two patients showed remission of NCPH or portal obstruction/stenosis after receiving immunosuppression and anticoagulant therapy, both with ANCA-associated vasculitis (12). It suggests that treatment and prognosis seem to differ among patients with different type of vasculitis. In the current case, oral prednisone administration relieved non-specific symptoms such as fever and fatigue. However, PHT-related symptoms caused by severe portal venous stenosis still progressed even with immunosuppressive medication. Portal venous angioplasty with stent placement has direct effectiveness in allowing blood flow into the liver and decreasing portal venous pressure, thereby relieving the symptoms caused by PHT (22). Therefore, Portal venous angioplasty with stent placement is a practical and effective treatment to prevent PHT-related complications and improve liver function in cases where drug therapy fails.

## Conclusion

PHT and hepatic lobar atrophy due to portal venous stenosis is an extremely rare manifestation of systemic vasculitis, which represents a diagnostic and therapeutic challenge. There is high predictive value of hepatic lobar atrophy in portal obstruction. CTA and portal venography further provide valuable evidence for the diagnosis of portal obstruction. Portal venous angioplasty with stent placement is a practical and effective treatment to prevent PHT-related complications when immunosuppression has failed.

## Data availability statement

The original contributions presented in the study are included in the article/supplementary material. Further inquiries can be directed to the corresponding authors.

## Ethics statement

Written informed consent was obtained from the individual(s) for the publication of any potentially identifiable images or data included in this article.

## Author contributions

We all provided care for the patient. We all devised and approved the final version of the manuscript. QC and YT produced the initial draft. BW provided the angiographic images. HW and YT reviewed and edited the manuscript.

## Funding

This work was supported by the National Natural Science Fund of China (Grant No. 82000574), Sichuan Science and Technology Program (Grant No. 2020YJ0084), 1·3·5 project for disciplines of excellence – Clinical Research Incubation Project (Grant No. 2019HXFH024), and Post-Doctor Research Project (Grant No. 2019HXBH074), West China Hospital, Sichuan University.

## Conflict of interest

The authors declare that the research was conducted in the absence of any commercial or financial relationships that could be construed as a potential conflict of interest.

## Publisher’s note

All claims expressed in this article are solely those of the authors and do not necessarily represent those of their affiliated organizations, or those of the publisher, the editors and the reviewers. Any product that may be evaluated in this article, or claim that may be made by its manufacturer, is not guaranteed or endorsed by the publisher.
